# Associations of safe nurse staffing coverage and care complexity factors with nursing-sensitive adverse events in cardiac inpatients: a cross-sectional multicenter study

**DOI:** 10.3389/fcvm.2026.1810207

**Published:** 2026-06-12

**Authors:** Susana Asensio-Flores, Jordi Adamuz, Rosa Soldevila-Cases, Mònica Castellà-Creus, Esperanza Zuriguel-Perez, Oliver Polushkina-Merchanskaya, Pilar Delgado-Hito, Maria-Eulàlia Juvé-Udina

**Affiliations:** 1 Hospital Universitari de Bellvitge, Bellvitge Biomedical Research Institute (IDIBELL), Barcelona, Spain; 2 Faculty of Nursing, University of Barcelona, L’Hospitalet de Llobregat, Barcelona, Spain; 3 Nursing Knowledge Management and Information Systems Department, Hospital Universitari de Bellvitge, Bellvitge Biomedical Research Institute (IDIBELL), L’Hospitalet de Llobregat, Barcelona, Spain; 4 Hospital Universitari de Bellvitge, L’Hospitalet de Llobregat, Barcelona, Spain; 5 Quality Department, Hospital Germans Trias I Pujol, Barcelona, Spain; 6 Multidisciplinary Nursing Research Group (VHIR), Knowledge Management and Evaluation, Hospital Universitari Vall d’Hebron, Barcelona, Spain; 7 Nursing Research Group, Bellvitge Biomedical Research Institute (IDIBELL), L’Hospitalet del Llobregat, Catalonia, Spain; 8 Catalan Health Institute, Bellvitge Biomedical Research Institute (IDIBELL), L’Hospitalet de Llobregat, Barcelona, Spain

**Keywords:** adverse events, care complexity factors, nurse staffing, patient outcome, quality of health care

## Abstract

**Background:**

Adverse events (AEs) are linked to nurse staffing levels and care complexity factors; however, evidence in patients hospitalized for cardiac conditions is limited. The acute to intensive care (ATIC) patient classification system measures patient acuity according to required nurse hours per patient day (NHPPD), thereby enabling calculation of nurse staffing coverage. A prior study defined safe nurse staffing coverage as achieving ≥90% of the required NHPPD with the available nurse staffing. The aim of this study was to examine the associations between safe nurse staffing coverage and care complexity factors and AEs in patients hospitalized for cardiac conditions in three high-complexity hospitals in Catalonia between January and December 2022.

**Methods:**

This was a cross-sectional multicenter study that included patients admitted for cardiological reasons at three high-complexity hospitals of the Catalan Institute of Health in Catalonia between January and December 2022. Data regarding care complexity individual factors and patient acuity, according to the ATIC patient classification system, were obtained from electronic health records. The main outcomes were nursing-sensitive AEs that included falls, pressure ulcers, phlebitis, cardiorespiratory arrest, acute pulmonary edema (APE), and aspiration pneumonia. Safe nurse staffing coverage was defined as achieving ≥90% of the required NHPPD. Other clinical and sociodemographic variables were also collected. A descriptive and comparative analysis was performed.

**Results:**

Among the 6,873 patients included in the study, 11.4% experienced one of the AEs studied, the most common being APE, present in 5% of cases. The mean nurse staffing coverage was 64.3%. Patients admitted to step-down units and those admitted for surgical reasons received the highest nurse staffing coverage, at 97.7% and 65.7%, respectively. The risk factors independently associated with AEs were old age, consciousness disorders, chronic disease, hemodynamic instability, and transmissible infection. Safe nurse staffing coverage was a protective factor against AEs.

**Conclusions:**

Older age, consciousness disorders, chronic disease, hemodynamic instability, and transmissible infection were independently associated with nursing-sensitive AEs, while safe nurse staffing coverage was a protective factor. These findings should inform staffing decisions and shape future healthcare-management policies aimed at improving health outcomes among adults hospitalized in cardiology wards.

## 1 Justification/Introduction

Adverse events (AEs) remain a major challenge to patient safety. The World Health Organization (WHO) defines patient safety as the reduction of preventable harm associated with healthcare to an acceptable minimum. In this context, AEs are harmful incidents associated with healthcare and include medication errors, healthcare-associated infections, diagnostic errors, patient falls, and pressure ulcers (1). The Seventy-Fourth World Health Assembly (2021) estimated that, in high-income countries, approximately one in 10 patients experiences an AE, and that the social cost of patient harm may reach US $1–2 trillion annually (2). Consistent with this, a systematic review by Sauro et al. (3) across 94 studies, representing 590 million patients, reported an overall hospital AE rate of 8.6 per 100 patient admissions, with more than half of the AEs reported as preventable (52.6%). In Spain, the ENEAS study included 1,063 patients, from 24 hospitals, who experienced an adverse event. The incidence of AEs associated with hospital assistance was 8.4% (4). Regarding cardiological patients, a cross-sectional study found a 16.3% AE rate, including drug-related, operation-related, and procedure-related AEs, at a teaching hospital in Japan (5).

Several factors have been linked to AEs in hospitalized patients, such as age, comorbidities, length of stay, and other care complexity factors (3, 6). Other studies have identified organizational factors, particularly nurse staffing levels, nursing skill mix, and nursing team composition, as factors associated with health outcomes, including mortality, hospital-acquired infections, and overall length of stay (7–10). However, approaches to determining nurse staffing levels are heterogeneous; the majority of studies refer to the number of nurse hours per patient day (NHPPD) or per shift. Others estimated the number of nurse hours above or below the expected mean (10, 11); however, only a limited number of studies have accounted for the totality of patients’ care needs when estimating nurse staffing requirements (12).

In recent years, different patient classification systems have been developed. Some enable the number of hours of nursing care needed for every patient to be determined using acuity measurement tools. The acute to intensive care (ATIC) patient classification system, based on the weight of the patient's main symptom, is structured into 10 acuity groups and their equivalence to required nursing hours per patient per day (rNHPPD) (13). The balance between rNHPPD and available nursing hours per patient per day (aNHPPD) is calculated as the difference between these two measures and is translated into a percentage of nurse staffing coverage (NSC), that is, the proportion of rNHPPD needed to meet the patient's safety needs covered by the aNHPPD (12). According to a previous study, the threshold for patient safety is approximately 90% (14). This is consistent with Park's sweet spot theory-driven implementation strategy, presented as an intersectional “optimum nurse staffing zone” in multiple given models (15).

Although the impact of nursing coverage on the health outcomes of patients hospitalized in Catalonia has been studied across all specialties (12, 14), evidence specific to cardiac admissions remains scarce. Moreover, while numerous clinical predictors of AEs have been described in cardiology inpatients (5, 16), far fewer studies have considered broader health determinants such as care complexity individual factors (CCIFs), patient acuity, or safe nursing staffing (17). In this sense, evidence suggests that developmental, emotional, mental-cognitive, and sociocultural complexity factors, as well as those related to comorbidities, are associated with AEs (18). However, although a previous study explored CCIFs among cardiology patients (19), the specific impact of safe nurse staffing coverage and CCIFs on AEs in patients hospitalized for cardiac conditions remains unclear.

Therefore, this study aimed to examine whether safe nurse staffing coverage and care complexity factors are associated with AEs among patients hospitalized for cardiac conditions at three high-complexity hospitals.

## 2 Methods

### 2.1 Setting and study design

This was a cross-sectional study that was conducted at three high-tech public hospitals in Spain from 1 January to 31 December 2022.

### 2.2 Participants

All adults admitted to the cardiology and cardiac surgery departments, in both acute and step-down units, were included. Participants had to be 18 years of age or older.

### 2.3 Ethical considerations

This study was conducted in accordance with the ethical standards set forth in the Declaration of Helsinki and was evaluated and approved by the Clinical Research Ethics Committee of Bellvitge University Hospital (reference PR368/21). The retrospective nature of the study meant that informed consent was not necessary. All data were anonymized using a unique identification number, so anonymity and data confidentiality guidelines were ethically complied with throughout the study.

#### 2.3.1 Patient and public involvement

Patients and/or the public were not involved in the design, conduct, reporting, or dissemination of the plans of this research.

### 2.4 Variables

Nurse staffing measures were defined as previously described by Juvé-Udina et al. (13); aNHPPD were calculated by dividing the available registered nurse hours by the total number of patients in each unit each day. Patient counts were aggregated by shift and day, according to unit assignment reports.

rNHPPD were determined based on the main nursing diagnoses identified in the nursing records, using the ATIC patient classification system. This system divides patient acuity into 10 categories of nursing intensity, which correspond to the rNHPPD (13).

NSC was calculated as the difference between rNHPPD and aNHPPD, expressed as a percentage.

In this study, safe nurse staffing coverage was defined as ≥90%, following prior work by Juvé-Udina et al., which identified this threshold as associated with a lower incidence of poor patient outcomes (14).

Nurse-sensitive AEs: Based on a previous study (18), we selected nurse-sensitive outcomes that are relevant to cardiology inpatients. Accordingly, we included the following nurse-sensitive AEs: falls, pressure ulcers, phlebitis, cardiorespiratory arrest, acute pulmonary edema (APE), and aspiration pneumonia. Only AEs occurring during hospitalization were included. Nurse-sensitive AEs were coded as dichotomous patient-level variables, indicating whether each patient experienced at least one AE during hospitalization, regardless of the number of events recorded. AEs were identified using the International Classification of Diseases, 10th revision (ICD-10) codes recorded in the electronic health record systems and extracted from the hospitals’ minimum data sets (see Supplementary File S1).

Care complexity individual factors: The 28 CCIFs were classified into the following five domains as previously described by Juvé-Udina et al. (20): developmental, mental-cognitive, psycho-emotional, sociocultural, and comorbidity/complications. The developmental domain included one factor: old age (≥75 years). The mental-cognitive domain included four factors: (i) agitation; (ii) consciousness disorders (confusion, disorientation, stupor, or transient loss of consciousness); (iii) impaired cognitive functions (intellectual disability or amnesia); and (iv) perception of reality disorders (delirium, hallucinations, or disconnection from reality). The psycho-emotional domain comprised the following three factors: (i) aggressive behavior; (ii) fear/anxiety; and (iii) impaired adaptation (disruptive behavior, hopelessness, or surrender). The sociocultural domain included five factors: (i) language barriers, (ii) social exclusion (extreme poverty), (iii) belief conflict (spiritual distress), (iv) illiteracy, and (v) lack of caregiver support. Finally, the comorbidity/complications domain contained the following 15 factors: (i) major chronic disease; (ii) hemodynamic instability (intensive control of vital signs or state of shock); (iii) high risk of hemorrhage (coagulation disorders, thrombocytopenia, or anticoagulant therapy); (iv) communication disorders (aphasia, dysphasia, dysarthria, laryngectomy, or tracheostomy); (v) urinary or fecal incontinence; (vi) vascular fragility (capillary fragility or tortuous veins); (vii) position impairment; (viii) involuntary movements (continuous involuntary movements); (ix) extreme weight (low weight or obesity); (x) dehydration (skin turgor); (xi) edema; (xii) uncontrolled pain (verbal numerical rating scale above three points); (xiii) transmissible infections (isolation measures); (xiv) immunosuppression; and (xv) anatomical and functional disorders (amputation, deformities, or joint stiffness). CCIFs were identified through clinical manifestations recorded in the nursing assessment using ATIC terminology, which has been used in all the hospitals in the Catalan Institute of Health since 2007.

We also recorded other clinical variables, namely reason for admission (medical or surgical reason for admission) and unit type (acute care or step-down unit), and the following sociodemographic variables: age, sex, length of stay, and admission to intensive care unit (ICU).

### 2.5 Data collection

All data were collected retrospectively from the electronic health record systems, the hospitals’ minimum data sets, and the clinical data repository of the Catalan Institute of Health. All patient data collected were blinded using consecutive participant numbers that were used to link the data sets from these sources. Nurse staffing data were obtained from human resources databases and ward structural assignment reports.

### 2.6 Statistical analysis

A descriptive analysis of sociodemographic variables, care complexity individual factors, and the incidence of AEs was performed. Continuous variables that followed a normal distribution are presented as mean and standard deviation, continuous variables that did not follow a normal distribution are presented as median and interquartile range, and categorical variables are presented as number of cases and percentage.

A comparative analysis of the level of nurse staffing coverage was performed by unit setting and reason for admission, as well as between patients who had experienced an AE and those who had not. Percentage differences among unit-clusters were analyzed using the chi-square test for categorical variables, while for continuous variables, we used Student's *t*-test or the Mann–Whitney *U*-test, depending on the results of the Kolmogorov–Smirnov normality test.

A multivariate logistic regression was performed for safe nurse staffing coverage (≥90% of nurse staffing coverage) and CCIFs. We included all the significant and clinically relevant CCIFs detected in the univariate analysis. This analysis adjusted for the unit setting, reason for admission, average length of stay, ICU admission, and sex.

Data analyses were performed using R version 4.4.1. (R Core Team 2025). *P*-values less than 0.05 were considered statistically significant.

## 3 Results

**Figure 1:**
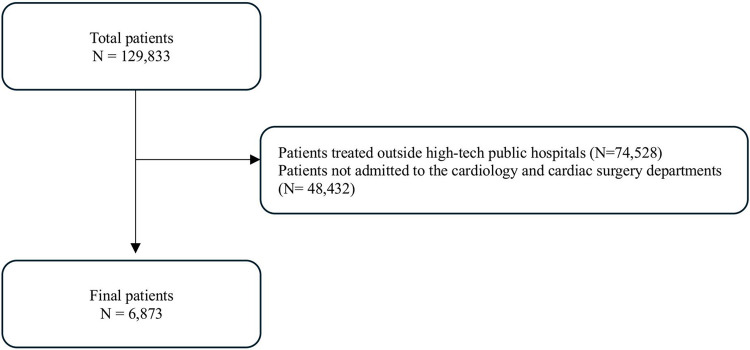
Flowchart of the included patients.

A total of 6,873 patients were included in the study (Figure 1); 64.2% (4,411) were men, and the mean age was 67.5 years (SD 14.07). A total of 39.2% (*n* = 2,693) of the patients had been admitted to the ICU, and the mean length of stay for the patient sample was 9.18 days (SD 13.06). Moreover, 75.8% had been admitted to surgical wards and 20.1% to step-down units. The most common admission diagnoses were ischemic heart disease (26.9%), cardiac surgery (19.3%), and pacemaker/defibrillator (11.7%) (Table 1).

**Table 1:** Baseline characteristics of the included adult patients.

Main characteristics	*N*	(%)
Demographic characteristics		
Age (years)_mean (SD)	67.5	(14.07)
Age ≥ 75 years	2,441	(35.5)
Male sex	4,411	(64.2)
Prior ICU admission	2,693	(39.2)
Discharge to another facility	364	(5.4)
Length of stay (days)_mean (SD)	9.18	(13.06)
Surgical ward	5,210	(75.8)
Step-down unit	1,384	(20.1)
Underlying disease		
Chronic heart disease	2,228	(32.4)
Chronic renal disease	1,213	(17.6)
Chronic respiratory disease	873	(12.7)
Chronic liver disease	312	(4.5)
Neurodegenerative disease	38	(0.6)
Admission diagnosis according to nursing care plans		
Ischemic heart disease	1,847	(26.9)
Cardiac surgery	1,325	(19.3)
Pacemaker/defibrillator	804	(11.7)
Cardiac rhythm and conduction disorders	648	(9.4)
Heart failure	473	(6.9)
Electrophysiological study	352	(5.1)
Coronary and structural interventional procedures	748	(10.9)
Cardiac valve diseases	160	(2.3)
Presurgical/interventional preparation	125	(1.8)
Pericardial diseases	74	(1.1)

SD, standard deviation; ICU, intensive care unit.

Of the total number of patients studied, 11.4% (*n* = 782) experienced at least one adverse event, the most common being APE, present in 5% (*n* = 345) of cases, followed by phlebitis 4.4% (*n* = 305), cardiac arrest 0.9% (*n* = 65), and falls 0.9% (*n* = 60) (Table 2).

**Table 2:** Number of nursing-sensitive adverse events among the included patients.

Nursing sensitive adverse events	*N*	(%)
No. of adverse events	782	(11.4)
Acute pulmonary edema	345	(5.0)
Phlebitis	305	(4.4)
Cardiac arrest	65	(0.9)
Fall	60	(0.9)
Pressure ulcer	58	(0.8)
Aspiration pneumonia	27	(0.4)

### 3.1 Care complexity individual factors and nurse staffing measures according to AE

Regarding the CCIFs, 92.2% of the patients presented with complexity factors in the comorbidity/complications domain, with the most notable factors being hemodynamic instability [84.3% (*n* = 5,793)], major chronic disease [45% (*n* = 3,096)], and uncontrolled pain [20.2% (*n* = 1,389)]. In the developmental domain, 35.5% (*n* = 2,441) presented with old age (>75 years). Regarding the mental-cognitive domain, 7.8% (*n* = 539) of the patients presented with consciousness disorders. Finally, in the psycho-emotional domain, 5.7% (*n* = 393) of the patients presented with fear/anxiety and 3.2% with impaired adaptation (*n* = 220). Thus, the included patients had an average of 2.4 CCIFs (SD 1.49). Moreover, the univariate analysis showed that the average number of CCIFs was higher among the patients with AEs (3.15 vs. 2.34, *p* < 0.001).

Regarding nurse staffing measures, the mean number of nursing hours required by patients was 5.1 rNHPPD (SD 1.6), the mean number of available nursing hours was 2.98 aNHPPD (SD 0.99), and the mean nurse staffing coverage was 64.3% (SD 33.7). Moreover, the patients who did not experience AEs received higher coverage than those who did, with a statistically significant difference of approximately 10 percentage points between groups (55.2% SD 23.1 vs. 65.53% SD 34.7), *p* < 0.001. Finally, safe nurse staffing (≥90%) was significantly less frequent among patients with AEs than among those without AEs (7.2% vs. 14.2%; *p* < 0.001) (Table 3).

**Table 3:** Clinical characteristics, CCIFs, and nurse staffing measures according to nursing-sensitive adverse events.

Clinical characteristics, CCIFs, and nurse staffing measures	Total		AEs		No AEs		*p*-Value
	*n* = 6,873		*n* = 782 (11.4)		*n* = 6,091		
	*N*	(%)	*N*	%	*N*	%	
Clinical characteristics							
Age (years)_mean (SD)	67.5	(14.07)	69.80	(12.91)	62.20	(14.19)	<0.001
Age ≥ 75 years	2,441	(35.5)	328	(41.9)	2,113	(34.7)	<0.001
Male sex	4,411	(64.2)	484	(61.9)	3,927	(64.5)	0.085
Prior ICU admission	2,693	(39.2)	468	(59.8)	2,225	(36.5)	<0.001
Discharge to another facility	364	(5.4)	100	(12.8)	268	(4.4)	<0.001
Length of stay (days)_mean (SD)	9.18	(13.06)	19,7	(24.75)	7.83	(9.90)	<0.001
Reason for admission							0.723
Surgical	5.210	(75.8)	597	(76.3)	4,613	(75.7)	
Medical	1,663	(24.2)	185	(23.7)	1,478	(24.3)	
Unit setting							0.012
Step-down unit	1,384	(20.1)	131	(16.8)	1,253	(20.6)	
Acute	5,489	(79.9)	651	(83.2)	4,838	(79.4)	
CCIFs							
Comorbidity/complications	6,334	(92.2)	762	(97.4)	5,572	(91.5)	<0.001
Hemodynamic instability	5,793	(84.3)	718	(91.8)	5,075	(83.3)	<0.001
Major chronic disease	3,096	(45.0)	478	(61.1)	2,618	(43.0)	<0.001
Uncontrolled pain	1,389	(20.2)	233	(29.8)	1,156	(19.0)	<0.001
Extreme weight	696	(10.1)	99	(12.7)	597	(9.8)	0.017
Edema	606	(8.8)	98	(12.5)	508	(8.3)	<0.001
Mixed incontinence	394	(5.7)	70	(9.0)	324	(5.3)	<0.001
Anatomical and functional disorders	278	(4.0)	49	(6.3)	229	(3.8)	0.001
Transmissible infection	276	(4.0)	71	(9.1)	205	(3.4)	<0.001
Position impairment	174	(2.5)	41	(5.2)	133	(2.2)	<0.001
Vascular fragility	101	(1.5)	14	(1.8)	87	(1.4)	0.429
Communication disorders	55	(0.8)	16	(2.0)	39	(0.6)	<0.001
High risk of hemorrhage	27	(0.4)	6	(0.8)	21	(0.3)	0.117
Immunosuppression	13	(0.2)	0	(0.0)	13	(0.2)	0.385
Involuntary movements	5	(0.1)	1	(0.1)	4	(0.1)	0.453
Dehydration	1	(0.0)	0	(0.0)	1	(0.0)	–
Developmental	2,449	(35.6)	328	(41.9)	2,121	(34.8)	<0.001
Old age (≥75 years)	2,441	(35.5)	328	(41.9)	2,113	(34.7)	<0.001
Psycho-emotional	597	(8.7)	78	−10	519	(8.5)	0.177
Fear/anxiety	393	(5.7)	57	(7.3)	336	(5.5)	0.049
Impaired adaptation	220	(3.2)	21	(2.7)	199	(3.3)	0.450
Aggressive behavior	8	(0.1)	3	(0.4)	5	(0.1)	0.053
Mental-cognitive	553	(8.0)	136	(17.4)	417	(6.8)	<0.001
Consciousness disorders	539	(7.8)	133	(17.0)	406	(6.7)	<0.001
Agitation	25	(0.4)	4	(0.5)	21	(0.3)	0.520
Perception of reality disorders	19	(0.3)	2	(0.3)	17	(0.3)	0.631
Impaired cognitive functions	7	(0.1)	2	(0.3)	5	(0.1)	0.185
Sociocultural	123	(1.8)	16	(2.0)	107	(1.8)	0,566
Language barriers	82	(1.2)	7	(0.9)	75	(1.2)	0.488
Lack of caregiver support	40	(0.6)	11	(1.4)	29	(0.5)	0.004
Illiteracy	3	(0.0)	1	(0.1)	2	(0.0)	0.304
Social exclusion	1	(0.0)	0	(0.0)	1	(0.0)	–
Belief conflict	1	(0.0)	0	(0.0)	1	(0.0)	–
CCIF, mean, (SD)	2.43	(1.49)	3.15	(1.65)	2.34	(1.44)	<0.001
Nurse staffing measures							
rNHPPD_mean (SD)	5.10	(1.60)	5.48	(1.56)	5.05	(1.60)	<0.001
aNHPPD_mean (SD)	2.98	(0.99)	2,81	(0.88)	2.96	(1.00)	0.001
Balance_ mean (SD)	−2.15	(1.84)	−2.67	(1.76)	−2.08	(1.84)	<0.001
Coverage %_ mean (SD)	64.3	(33.7)	55.19	(23.09)	65.53	(34.68)	<0.001
Safe nursing staffing coverage (≥90%)	921	(13.4)	56	(7.2)	865	(14.2)	<0.001

CCIF, care complexity individual factors; SD, standard deviation; ICU, intensive care unit; rNHPPD, required nursing hours per patient day; aNHPPD, available nursing hours per patient day; AEs, adverse events.

### 3.2 Multivariate associations of safe nurse staffing coverage and CCIFs with AEs

**Figure 2:**
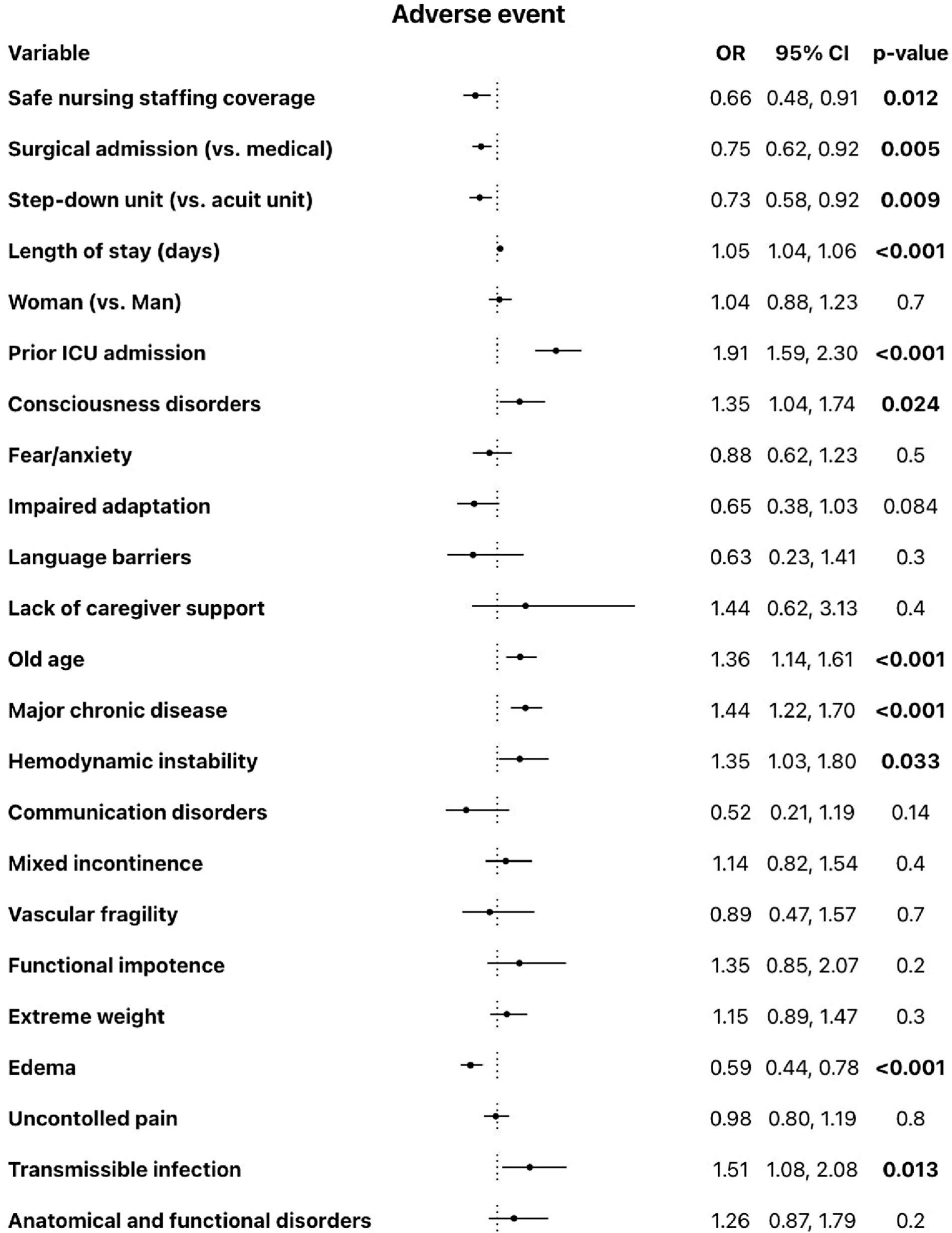
Forest plot of the multivariate-adjusted associations between safe nurse staffing coverage and care complexity individual factors, and nursing-sensitive AEs.

The multivariate analysis showed that safe nursing coverage (OR 0.66; 95% CI 0.48–0.91) was a protective factor for AEs. Regarding CCIFs, old age (OR 1.36; 95% CI 1.14–1.61), major chronic disease (OR 1.44; 95% CI 1.22–1.70), consciousness disorders (OR 1.35; CI 95% 1.04–1.74), hemodynamic instability (OR 1.35; CI 95% 1.03–1.80), and transmissible infection (OR 1.51; CI 95% 1.08–2.08) were independent factors associated with AEs (Figure 2).

Moreover, admission for surgical reasons (OR 0.75; 95% CI 0.62–0.92) and admission to a step-down unit (OR = 0.73; 95% CI 0.58–0.92) were protective factors against the onset of AEs (*p* < 0.05), while length of stay (OR 1.05; 95% CI 1.04–1.06) and prior ICU admission (OR 1.91; 95% CI 1.59–2.39) were risk factors for experiencing AEs.

### 3.3 Nurse staffing measures according to admission setting and reason of admission

A *post-hoc* analysis was performed to describe nurse staffing measures according to admission setting and reason for admission (Table 4). The mean number of required nursing hours was higher in step-down units (5.2 rNHPPD; SD 1.4) and among patients admitted for medical reasons (5.5 rNHPPD; SD 1.7). In contrast, available nursing hours were higher among patients admitted for surgical reasons (3.0 aNHPPD; SD 1.0) and among those admitted to step-down units (4.6 aNHPPD; SD 0.9). Accordingly, patients admitted to step-down units and those admitted for surgical reasons showed the highest nurse staffing coverage, at 97.7% (SD 45.4) and 65.7% (SD 31.2), respectively.

**Table 4:** Nurse staffing measures according to admission setting and reason for admission.

Nurse staffing measures	All		Acute unit		Step-down unit		Medical		Surgical	
	*n* = 6,873		*n* = 5,489 (79.9)		*n* = 1,384 (20.1)		*n* = 1,663 (24.2)		*n* = 5,210 (75.8)	
	*N*	(%)	I	(%)	*N*	(%)	*N*	(%)	*N*	(%)
rNHPPD_mean (SD)	5.10	(1.60)	5.08	(1.64)	5.17	(1.41)	5.49	(1.7)	4.97	(1.5)
*a*NHPPD_mean (SD)	2.98	(0.99)	2.54	(0.46)	4.56	(0.89)	2.81	(0.7)	2.99	(1.05)
Balance_ mean (SD)	−2.15	(1.84)	−2.54	(1.66)	−0.61	(1.70)	−2.67	(1.9)	−1.98	(1.7)
Coverage %_ mean (SD)	64.3	(33.7)	55.9	(23.4)	97.7	(45.4)	60	(40.1)	65.7	(31.2)

SD, standard deviation; ICU, intensive care unit; rNHPPD, required nursing hours per patient day; aNHPPD, available nursing hours per patient day.

## 4 Discussion

This study shows that 11.4% of patients hospitalized for cardiac reasons experienced AEs during their stay. The average nurse staffing coverage was 64.3%. Patients admitted to step-down units and those admitted for surgical reasons received the highest nurse staffing coverage, at 97.7% and 65.7%, respectively. The risk factors independently associated with AEs were old age, consciousness disorders, chronic disease, hemodynamic instability, and transmissible infection. Safe nurse staffing coverage was a protective factor against AEs.

The incidence of AEs was higher than that reported in the ENEAS study in Spain (4). This is also consistent with the findings of a meta-analysis by Sauro et al. (3), which concluded that the overall pooled estimate of hospital AEs was 8.6 per 100 patient admissions. In our study, we found that the incidence was lower than that reported in another study on the incidence of AEs in cardiology patients (5). However, the majority of studies on AEs report those related to invasive procedures, administered medications, or misdiagnosis (16, 21).

The mean nurse staffing coverage in the study population was 64.3%. This coverage is higher than that detected in a study by Adamuz et al. (17) in patients admitted with COVID-19 (40.9%) but similar to that observed in adult patients admitted by medical and surgical services, excluding maternal and child care (70.9% and 65.5%, respectively) (14, 22). Patients admitted to step-down units and those admitted for surgical reasons received the highest nurse staffing coverage (97.7% and 65.7%, respectively). These results may be related to the profile of patients admitted to step-down units, as they typically require more nursing care. Traditionally, the nurse-to-patient ratio in these units is maintained at 1:4 across all shifts, whereas in acute wards, this ratio may vary between different hospitals and between day and night shifts (14). However, despite the relevance of patient needs in determining staffing requirements, evidence suggests that neither patient status nor level of care dependency is routinely incorporated into day-to-day nurse staffing planning (23).

Historically, analyses of the nursing care required by surgical patients, sometimes based on the concept of workload, have led to a higher number of nurses working in surgical units than in medical ones. Although recent studies have shown that patients admitted to medical units may actually have the highest acuity profiles (24), registered nurse understaffing in the first 6 postoperative days has been associated with higher odds of postoperative atrial fibrillation (25). In this context, recent evidence also supports the value of systematic nursing assessment on admission and throughout hospitalization to identify patients at increased risk of adverse outcomes, including in-hospital mortality (26).

The main finding of this research is that safe nurse staffing coverage (≥90%) is associated with a significant reduction in the risk of AEs. The odds ratio (OR) values in the adjusted regression model indicate that safe nurse staffing coverage acts as a protective factor against adverse outcomes. These results align with those obtained in hospitalized patients (14) and admitted patients with COVID-19 (17). In these studies, a high level of nurse staffing coverage protected against the onset of AEs and in-hospital mortality.

The multivariate analysis showed that safe nurse staffing coverage was a protective factor against AEs, as was admission to step-down or surgical units. Considering that these settings typically have higher levels of nurse staffing coverage (12), this finding further supports the impact of nurse staffing coverage on patient outcomes.

In addition, patients admitted to hospitals for cardiac reasons had an average of two individual complexity factors. This finding is consistent with a previous study in a comparable cardiac cohort, which also reported a mean of two CCIFs (19). Likewise, among admissions to Catalan hospitals across all specialties, a median of two CCIFs (IQR 1–3) has been documented (6, 18). Therefore, patients admitted for cardiac conditions exhibit a level of care complexity comparable to that of the overall hospitalized population.

Finally, our study found that prolonged length of stay, previous ICU admission, old age, and the presence of comorbidities were risk factors for the development of AEs, as identified in previous studies (27). In addition, consciousness disorders, hemodynamic instability, and transmissible infection were independently associated with experiencing AEs. Overall, these findings support that care complexity individual factors are linked to a higher incidence of AEs, in line with prior studies (18, 22, 28). Therefore, both safe nurse staffing levels and care complexity factors should be considered in future policies to optimize nurse staffing levels in cardiology units. In line with the WHO Global Patient Safety Action Plan 2021–2030, these findings highlight the need for health systems and healthcare organizations to strengthen their capacity to identify patient safety risks and address avoidable sources of harm (1).

This study has several strengths, including its multicenter observational cohort design and the large sample size. To the best of our knowledge, it is the first study to assess the impact of safe nurse staffing and CCIFs on AEs in admitted cardiology patients. However, some limitations should be acknowledged. All data were extracted from electronic health records, which included nursing assessments at admission and during hospitalization. As such, we assumed adequate compliance with documentation standards. Nevertheless, the possibility of coding errors cannot be excluded. We believe, however, that the large sample size likely mitigates the impact of such potential inaccuracies. Furthermore, some determinants of health outcomes, including the nursing skill mix and the composition of the nursing team, were not incorporated into this study. Future research in this area should aim to integrate these variables to provide a more comprehensive analysis. Finally, given the cross-sectional design, inferences are associative rather than predictive or causal; prospective studies are needed to test whether achieving ≥90% coverage reduces AEs.

## 5 Conclusions

Older age, consciousness disorders, chronic disease, hemodynamic instability, and transmissible infection were independently associated with AEs in patients hospitalized for cardiac conditions, whereas nurse staffing coverage ≥90% acted as a protective factor against nursing-sensitive AEs. These findings should inform staffing decisions and healthcare management policies by reinforcing the need to align human resource planning with patient acuity and care demands, in line with the WHO’s vision of safer, more respectful care for every patient.

## Data Availability

All relevant data are available in the article or the Supplementary Materials. Requests to access the datasets should be directed to jadamuz@bellvitgehospital.cat.
