# Supplementary material for: Associations of safe nurse staffing coverage and care complexity factors with nursing-sensitive adverse events in cardiac inpatients: a cross-sectional multicenter study
Source: Front Cardiovasc Med. 2026 Jun 12;13:1810207. doi: 10.3389/fcvm.2026.1810207 (PMC13303206; doi:10.3389/fcvm.2026.1810207)
Supplement: Supplementary file 1 [file Table1.docx]

| **Supplementary file 1. Detailed ICD-10 and ATIC v5.3.9 codes for nursing- sensitive AEs.** | | |
| --- | --- | --- |
| ***Adverse event*** | **ICD-10 Code^a^** | **ICD-10 Label** |
| Aspiration pneumonia | J69.0  J69.8 | Pneumonitis due to inhalation food or vomitus  Pneumonitis due to inhalation of other solid and liquids |
| Cardiorespiratory arrest | I46.2  I46.8  I46.9  R09.2 | Cardiac arrest due to underlying cardiac condition  Cardiac arrest due to other underlying condition  Cardiac arrest, cause unspecified  Respiratory arrest |
| Acute pulmonary edema | J81.0 | Acute pulmonary edema |
| ***Adverse event*** | **ATIC v5.3.9 Code^a^** | **ATIC v5.3.9 Label** |
| Falls | 10000582  10007835  10006754  10007834  10006755  10006756  10007836  10008454 | Fall  Fall without visible injury  Fall with minor injury  Fall with multiple minor injuries  Fall with moderate injury  Fall with severe injury  Fall with temporary loss of awareness  Fall with multiple injuries |
| Pressure ulcers | 10004898  10006092  10006093  10004899  10006094  10006095  10004900  10006096  10006097  10004901  10006098  10006099  10008129 | Pressure ulcer stage 1  Pressure injury stage 1  Decubitus ulcer stage 1  Pressure ulcer stage 2  Pressure injury stage 2  Decubitus ulcer stage 2  Pressure ulcer stage 3  Pressure injury stage 3  Decubitus ulcer stage 3  Pressure ulcer stage 4  Pressure injury stage 4  Decubitus ulcer stage 4  Unstageable pressure ulcer |
| Venous catheter-related phlebitis | 10005869  10005870  10001284  10010467  10010468  10010469  10010470 | Infusion phlebitis  Drug perfusion phlebitis  Venous catheter-related phlebitis  Unknown origin phlebitis  Probable infectious origin  Probable chemical origin  Probable mechanical origin |
| ^a^ Not present on admission day. ICD-10 data were obtained from the hospitals minimum data set, as secondary diagnosis. All were checked for *not present on admission* criterion. | | |
